# Supplementary material for: Cortical D1 and D2 dopamine receptor availability modulate methylphenidate-induced changes in brain activity and functional connectivity
Source: Commun Biol. 2022 May 30;5:514. doi: 10.1038/s42003-022-03434-5 (PMC9151821; doi:10.1038/s42003-022-03434-5)
Supplement: Supplementary file 2 — Supplementary Information [file 42003_2022_3434_MOESM2_ESM.pdf]

SUPPLEMENTARY MATERIAL: Cortical D1 and D2 dopamine receptor availability modulate methylphenidate-induced changes in brain activity and functional connectivity

Supplementary Note 1. Additional models testing the relationship between Cortical D1R/D2R Ratio and age/spatial working memory performance. To determine whether sex and IQ were playing a critical role in the significant relationships between Age and D1R/D2R ratio (Association Cortices) and between spatial working memory and D1R/D2R ratio (Association Cortices) (**Figure 4, manuscript**), we performed additional linear regression models (using the *lm* function in R) between Age and D1/D2R and between spatial working memory and D1/D2R, including IQ and Sex as factors in the model. Neither factor was significant in either analysis (for the model with Age and D1D2R, the effect of sex: $t_{(35)} = -.185$, $p = .855$; and the effect of IQ: $t_{(35)} = 1.99$, $p = .055$; and for the model with spatial working memory and D1D2R, the effect of sex: $t_{(35)} = -.240$, $p = .812$; and the effect of IQ: $t_{(35)} = 1.500$, $p = .145$). In both models, the inclusion of these factors slightly reduced the strength of associations of Age and Spatial Working Memory with D1R/D2R ratio, which is not surprising since performance on an IQ test should share some variance with age and cognitive test performance: (For the association of D1D2R and Age: $t_{(35)} = -2.154$, $p = .039$; and for the association of D1D2R and spatial working memory: $t_{(35)} = -1.774$, $p = .086$).

Supplementary Figure 1. Correspondence between neocortical D2R availability assessed with [^{11}C]Raclopride and [^{18}F]Fallypride. For Raclopride, values were taken from the average of the 36 healthy adults used in the current study. For [^{18}F]Fallypride, values were taken from the average of 25 healthy adults used in a recently reported study with a publicly availably [^{18}F]Fallypride atlas (Castrellon et al., 2019). Datapoints reflect individual voxels (excluding all subcortical/cerebellar voxels). Despite the acknowledged weaker sensitivity of [^{11}C]Raclopride to detect extrastriatal D2 receptors, relative to [^{18}F]Fallypride (Svensson et al., 2019) there was moderate-to-strong correspondence between the two measures in neocortex (linear $r = .484$, quadratic $r = .759$). Further, when separating into sensorimotor and association networks, both tracers demonstrated a higher D2R availability in association compared to sensorimotor cortices (Association:Sensorimotor Ratio:[^{11}C]Raclopride = 1.07, [^{18}F]Fallypride = 1.42).

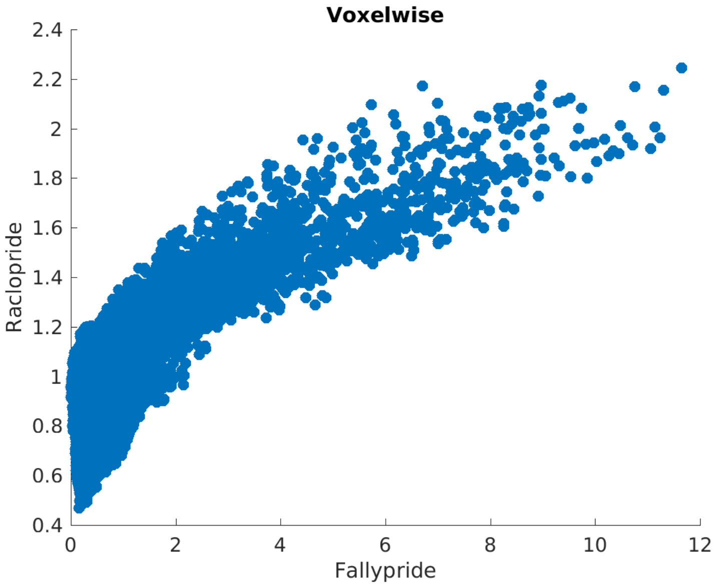
Voxelwise
Raclopride
Fallypride
2.4
2.2
2
1.8
1.6
1.4
1.2
1
0.8
0.6
0.4
0
2
4
6
8
10
12
