# Supplementary material for: Cortical D1 and D2 dopamine receptor availability modulate methylphenidate-induced changes in brain activity and functional connectivity
Source: Commun Biol. 2022 May 30;5:514. doi: 10.1038/s42003-022-03434-5 (PMC9151821; doi:10.1038/s42003-022-03434-5)
Supplement: Supplementary file 3 — Description of Additional Supplementary Files [file 42003_2022_3434_MOESM3_ESM.pdf]

Description of Additional Supplementary Files

File name: Supplementary Data 1
Description: Source data for figures 2, 3, and 4 from the manuscript.

File name: Supplementary Data 2
Description: Source data for Supplementary Figure 1.
