# Supplementary material for: Cortical D1 and D2 dopamine receptor availability modulate methylphenidate-induced changes in brain activity and functional connectivity
Source: Commun Biol. 2022 May 30;5:514. doi: 10.1038/s42003-022-03434-5 (PMC9151821; doi:10.1038/s42003-022-03434-5)
Supplement: Supplementary file 6 — Reporting Summary [file 42003_2022_3434_MOESM6_ESM.pdf]

nature research

Corresponding author(s): Peter Manza

Last updated by author(s): Mar 3, 2022

Reporting Summary

Nature Research wishes to improve the reproducibility of the work that we publish. This form provides structure for consistency and transparency in reporting. For further information on Nature Research policies, see our Editorial Policies and the Editorial Policy Checklist.

Statistics

For all statistical analyses, confirm that the following items are present in the figure legend, table legend, main text, or Methods section.

n/a	Confirmed	
☐	☒	The exact sample size (n) for each experimental group/condition, given as a discrete number and unit of measurement
☐	☒	A statement on whether measurements were taken from distinct samples or whether the same sample was measured repeatedly
☐	☒	The statistical test(s) used AND whether they are one- or two-sided<br>*Only common tests should be described solely by name; describe more complex techniques in the Methods section.*
☐	☒	A description of all covariates tested
☐	☒	A description of any assumptions or corrections, such as tests of normality and adjustment for multiple comparisons
☐	☒	A full description of the statistical parameters including central tendency (e.g. means) or other basic estimates (e.g. regression coefficient) AND variation (e.g. standard deviation) or associated estimates of uncertainty (e.g. confidence intervals)
☐	☒	For null hypothesis testing, the test statistic (e.g. F, t, r) with confidence intervals, effect sizes, degrees of freedom and P value noted<br>*Give P values as exact values whenever suitable.*
☒	☐	For Bayesian analysis, information on the choice of priors and Markov chain Monte Carlo settings
☒	☐	For hierarchical and complex designs, identification of the appropriate level for tests and full reporting of outcomes
☐	☒	Estimates of effect sizes (e.g. Cohen's d, Pearson's r), indicating how they were calculated

Our web collection on statistics for biologists contains articles on many of the points above.

Software and code

Policy information about availability of computer code

Data collection	PET scans were used to measure D1R availability with [11C]NNC-112 and to measure D2R availability with [11C]Raclopride. For each individual, studies were conducted on one of two scanners: a high-resolution research tomography (HRRT) scanner (n = 17; 7 female; Siemens AG; Germany) or a Biograph PET/CT scanner (n = 19; 6 females; Siemens AG; Germany). All subjects also underwent structural and resting-state functional MRI on a 3.0T Magnetom Prisma scanner (Siemens Medical Solutions USA, Inc., Malvern, PA) with a 32-channel head coil.
Data analysis	Analyses were performed in R version 3.6.2 and in GraphPad Prism version 8.0.1.<br>To test for regional differences in the PET measures (relative D1R, relative D2R, and relative D1R/D2R ratio), we perfomed paired t-tests (association versus sensorimotor).<br>To test for methylphenidate-induced changes in the fMRI measures (brain activity/connectivity), we perfomed paired t-tests (placebo vs. methylphenidate), each for association and sensorimotor cortices, and Bonferroni-corrected for two comparisons. Then, to see if the pattern of methylphenidate-induced changes in brain activity/connectivity differed by cortical regions, we performed a two-way repeated measures ANOVA, with drug (placebo versus methylphenidate) and network (association versus sensorimotor) as factors, and examined the interaction effect.<br>As a control analysis, we also examined traditional measures of striatal receptor availability. We tested for methylphenidate-induced changes in the D2R availability (i.e., 'dopamine increases') and tested whether D1R, D2R, D1/D2 ratio, and 'dopamine increases' correlated with baseline fMRI activity and connectivity, as well as methylphenidate-induced changes in activity and connectivity, both in sensorimotor and association cortices, using Pearson correlation.<br>Finally, we tested if the relative D1R/D2R ratio, each for association and sensorimotor cortices, was significantly associated with age and spatial working memory performance, using Pearson correlation. We hypothesized significant correlations would be observed in the association cortices, based on a large literature showing that dopamine receptor signaling in association regions such as prefrontal/parietal cortices is critical for spatial working memory 19. As an exploratory analysis, we repeated these tests in regression models that included sex and IQ as factors (using the lm function in R). Based on these tests, sex and IQ did not appear to play a major role in the results and these

additional models are presented in the Supplement.

For manuscripts utilizing custom algorithms or software that are central to the research but not yet described in published literature, software must be made available to editors and reviewers. We strongly encourage code deposition in a community repository (e.g. GitHub). See the Nature Research guidelines for submitting code & software for further information.

Data

Policy information about availability of data

All manuscripts must include a data availability statement. This statement should provide the following information, where applicable:

- Accession codes, unique identifiers, or web links for publicly available datasets
- A list of figures that have associated raw data
- A description of any restrictions on data availability

Summary data and R scripts used to produce primary results are in a publicly available repository: https://github.com/pmanza/Cortical-D1D2

Field-specific reporting

Please select the one below that is the best fit for your research. If you are not sure, read the appropriate sections before making your selection.

☒ Life sciences ☐ Behavioural & social sciences ☐ Ecological, evolutionary & environmental sciences

For a reference copy of the document with all sections, see nature.com/documents/nr-reporting-summary-flat.pdf

Life sciences study design

All studies must disclose on these points even when the disclosure is negative.

Sample size	36
Data exclusions	Due to poor image quality, fMRI data from one participant was removed. After this, fMRI analyses were performed twice: once with the entire sample (n = 35) and once after we removed participants with high levels of motion during resting fMRI (n = 5 removed due to >15% of timepoints 'scrubbed', reminder of particpants: n = 30). Since general findings did not change we report results from the full (n = 35) sample here.
Replication	Due to the costs associated with collecting PET data, it was not possible to replicate this study in a separate cohort.
Randomization	This is a within-subjects design. Participants were randomized to receive either methylphenidate or placebo on Day 1 vs. Day 2.
Blinding	Participants were blinded to the methylphenidate vs. placebo dose.

Reporting for specific materials, systems and methods

We require information from authors about some types of materials, experimental systems and methods used in many studies. Here, indicate whether each material, system or method listed is relevant to your study. If you are not sure if a list item applies to your research, read the appropriate section before selecting a response.

Materials & experimental systems

n/a	Involved in the study	
☒	☐	Antibodies
☒	☐	Eukaryotic cell lines
☒	☐	Palaeontology and archaeology
☒	☐	Animals and other organisms
☐	☒	Human research participants
☒	☐	Clinical data
☒	☐	Dual use research of concern

Methods

n/a	Involved in the study	
☒	☐	ChIP-seq
☒	☐	Flow cytometry
☐	☒	MRI-based neuroimaging

Human research participants

Policy information about studies involving human research participants

Population characteristics	Data from 36 healthy adults were included in the study (23 male, 13 female, age range 22-64) from the Washington DC metropolitan area.
Recruitment	Participants were recruited after initial screening via phone interview.

Ethics oversight | NIH Central IRB

Note that full information on the approval of the study protocol must also be provided in the manuscript.

Magnetic resonance imaging

Experimental design

Design type: Resting state

Design specifications: One per session (two sessions total); 8 minute acquisition time

Behavioral performance measures: None during MRI

Acquisition

Imaging type(s): functional

Field strength: 3T

Sequence & imaging parameters: A multi-echo, multiband EPI sequence was used: multiband factor = 3, anterior-posterior phase encoding, TR = 891 ms, echo times = 16, 33, and 48 ms, flip angle = 57 deg, 45 slices with 2.9 x 2.9 x 3.0 mm voxels and 520 time points

Area of acquisition: Whole-brain

Diffusion MRI: ☐ Used ☒ Not used

Preprocessing

Preprocessing software: SPM, FSL, AFNI

Normalization: Human Connectome Project functional pipeline

Normalization template: MNI

Noise and artifact removal: Signals from the white matter and CSF were regressed out of the data.

Volume censoring: Framewise displacements (FD) were computed from head translations and rotations using a 50 mm radius to convert angle rotations to displacements. Scrubbing was used to remove time points excessively contaminated with motion. Specifically, time points were excluded if the root mean square change in the BOLD signal (DVARS) from volume to volume met the criteria: DVARS > 0.5% and FD > 0.5 mm.

Statistical modeling & inference

Model type and settings: *Specify type (mass univariate, multivariate, RSA, predictive, etc.) and describe essential details of the model at the first and second levels (e.g. fixed, random or mixed effects; drift or auto-correlation).*

Effect(s) tested: To test for methylphenidate-induced changes in the fMRI measures (brain activity/connectivity), we perfomed paired t-tests (placebo vs. methylphenidate), each for association and sensorimotor cortices, and Bonferroni-corrected for two comparisons. Then, to see if the pattern of methylphenidate-induced changes in brain activity/connectivity differed by cortical regions, we performed a two-way repeated measures ANOVA, with drug (placebo versus methylphenidate) and network (association versus sensorimotor) as factors, and examined the interaction effect.

Specify type of analysis: ☐ Whole brain ☒ ROI-based ☐ Both

Anatomical location(s): Power Atlas

Statistic type for inference (See Eklund et al. 2016): N/A (no voxelwise analysis performed)

Correction: Bonferroni-corrected for two comparisons (association vs. sensorimotor cortices)

Models & analysis

n/a	Involved in the study
☒	☐ Functional and/or effective connectivity
☒	☐ Graph analysis
☒	☐ Multivariate modeling or predictive analysis
